# Natural experiments: A Nobel Prize awarded research design for strengthening causal inference on global health challenges

**DOI:** 10.7189/jogh.12.03005

**Published:** 2022-04-28

**Authors:** Famke JM Mölenberg, Francisca Vargas Lopes

**Affiliations:** 1 Department of Public Health, Erasmus MC, University Medical Center Rotterdam, Rotterdam, The Netherlands; 2 Erasmus Centre for Health Economics Rotterdam (EsCHER), Erasmus University Rotterdam, Rotterdam, The Netherlands

We would like to congratulate David Card, Joshua Angrist and Guido Imbens for winning the Nobel Memorial Prize in Economic Sciences 2021 for their pioneering work on natural experiments [1]. The committee acknowledged their work for “… shifting the focus in empirical research using observational data towards relying on quasi-experimental variation to establish causal effects.” On the occasion of their Nobel Prize, we would like to share a thought on learnings gained during our PhD trajectories in public health focused on natural experiments.

In public health, the opportunity of natural experiments to address global health challenges have been discussed for some years [2-5]. Natural experiments allow the retrospective and prospective evaluation of policies, interventions or programs in real-world settings [2]. Importantly, they present a valuable alternative to evaluate changes to a system for which it would be unethical, unfeasible or simply impossible to conduct randomised controlled trials (RCTs). Although there is not a widely accepted definition, the key element of natural experiments is that the change in exposure is caused by external shocks or factors outside researchers’ control, and that manipulation of exposure by researchers is not possible [2]. This allows the identification of intervention and control groups. While under ideal circumstances there is an “as-if” random allocation to the intervention, it is not uncommon that potential confounding remains in the effect of exposures on outcomes of interest. In combining good knowledge of the allocation process, careful choice of methods, and transparent reporting and assumption testing, studies based on natural experiments can approximate causal evidence [2].

It is likely that with this Nobel prize, opportunities for evaluations by means of natural experiments will be further explored. On a global scale, numerous of opportunities will arise from the sudden and disruptive changes linked to COVID-19 resulting from the global variation in national responses [6]. Therefore, it is important to understand the barriers to evaluate them. In this essay we build on learnings gained during our PhD trajectories focused on natural experiments. We discuss three key aspects hindering the potential of this type of research. We argue that, paradoxically, some level of control is needed to shape conditions in which evaluations of natural experiments is possible.

## THE CONVENIENCE OF UNPREDICTABILITY

There is considerable unpredictability in researching natural experiments, which may pose serious challenges for its evaluation. Unpredictability can be related to the implementation of the intervention (eg, timing, intensity and reach), but also to aspects related to the study conduction (eg, suitability of datasets and power). Studies using natural experiments in prospective evaluations may face difficulties aligning implementation, evaluation and funding timelines. For example, infrastructural interventions can be substantially delayed, while legislation is sometimes sooner implemented than anticipated; both impact heavily on timelines. Studies evaluating policies or interventions that have already been implemented will rely on previously collected data. This may sound like a secure route to minimise unpredictability, but exploratory data analysis is needed to assess whether assumptions and other statistical requirements of the study design are met. Not rarely, evaluations are altered or discontinued if evaluation in a meaningful way is not possible as expected when the natural experiment was identified. To overcome these challenges, researchers should be involved in early phases of intervention and policy planning, ensuring that key requirements to conduct evaluations through natural experiments are not missed. Based on our experience, early career researchers with relatively short contracts may benefit from joining existing collaborations with the fundaments for evaluation already present. Furthermore, research environments need to accommodate the intrinsic uncertainty of these studies. Providing the incentives to swiftly react on societal changes that suddenly occur are key: additional data can often be collected now, or never. For example, quick and flexible sources of funding have become available over the past months to study the COVID-19 pandemic. Similar initiatives are needed to combat big global health challenges that have been around for a while, including the “obesity epidemic”, the persistence of social inequalities, and the climate crisis.

**Figure:**
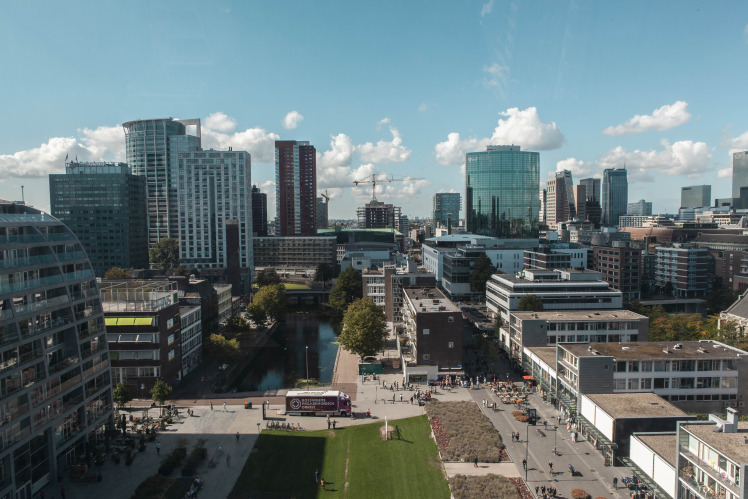
Photo: Natural experiments in cities allow evaluation of exposures that often cannot be randomised (Famke Mölenberg, personal collection, used with permission).

## THE CHALLENGE OF DATA LINKAGE

Even when data to evaluate the natural experiment are available, a complex factor is creating the database that includes all information needed. Linking datasets has been emphasised as an important aspect to foster the evaluation of natural experiments [7]. Even though databanks with linked administrative datasets are increasingly becoming available for entire countries or regions, some regions lack reliable data. Large secondary databases provide excellent opportunities to evaluate natural experiments based on already collected data, as long as researchers are sufficiently aware of their potential. Training on secondary data as formal part of research education might increase the opportunities for natural experiments evaluation. In absence of databanks, datasets need to be linked on a one-by-one basis. Informed consents – especially those that have been signed years ago – are often not designed to accommodate the linkage of datasets for the retrospective evaluation of natural experiments. Existing ethical and regulatory frameworks for sharing and processing personal data are sometimes subject for debate, making the alignment of different stakeholders a main barrier to proceed. Researchers need support by their own institutions to create multidisciplinary teams in which persons from various backgrounds (eg, legal officers, policymakers, practitioners, researchers) jointly facilitate the timely linkage of databases within ethical and regulatory frameworks.

## EMBRACING NATURAL EXPERIMENTS IN PUBLIC HEALTH

Natural experiments provide unique opportunities to strengthen the evidence base. To increase their adoption in public health, it is essential to improve the understanding of studies based on natural experiments. Over the years, we have received disappointing reviewer comments when submitting evaluations of natural experiments to public health journals. Some of the misunderstanding may result from different ways of conceptualising natural experiments [5]. Given the tendency in some journals to consider scientific rigor and associated uncertainties as more important than implications for professional practice, as well as the strict criteria for the use of causal language solely allowed for RCTs [8], means that the continuum of evidence from associations to causal conclusions is being ignored. Interdisciplinary research can be one way to learn from methodologies used by researchers in other fields [9]. Training of public health professionals, non-academic stakeholders, funders, and policymakers to understand the value and specificities of natural experiments is likely needed to increase the use of these evaluation strategies.

At last, we would like to draw attention to the career perspectives of researchers working on projects that capitalise on natural experiments. In a system where publications are still key to obtain new research funding and academic positions, evaluating small-scale interventions or conducting descriptive research might provide a more secure route to progress in academia [4]. This may pose a serious risk that the existing “evaluative bias” will increase, whereby most evidence is available for interventions that were easiest to study and to publish [10]. We need institutional changes where researchers are acknowledged for the societal relevance of their studies, not primarily on the quantity of publications. These risks are even larger for PhD candidates with strict timings and output objectives, possibly demotivating early career researchers from devoting their projects to evaluate natural experiments. Ultimately this may lead to less senior researchers being experts on natural experiments, and moving it forward in public health. Better understanding of the use and value is needed to ensure that brave researchers more often evaluate the interventions that hold large promise to inform decisions about population health.

In conclusion, natural experiments provide opportunities to inform policymaking on exposures that are impossible to randomise. While successful evaluations are available in literature, much can be learned from the barriers ultimately leading to unexplored opportunities, ceased projects, and unpublished manuscripts. As long as barriers are not addressed jointly by the research and policy environments, opportunities to provide evidence on global health challenges with extensive societal impact will continue to be missed.
